# Cultural Alignment and Psychological Well-Being: Insights from Person–Culture Match Research

**DOI:** 10.3390/healthcare14111513

**Published:** 2026-05-29

**Authors:** Vera Vogel

**Affiliations:** 1 GESIS—Leibniz Institute for the Social Sciences, 68159 Mannheim, Germany; vera.vogel@gesis.org; 2 School of Social Sciences, University of Mannheim, 68159 Mannheim, Germany

**Keywords:** person–culture match, person-environment fit, culture, well-being, mental health, public health implications

## Abstract

**Highlights:**

**What are the main findings?**

- Individual well-being is consistently higher when personal traits, values, or beliefs align with those prevalent in one’s sociocultural context, with effects spanning psychological health, physical health, and longevity.
- Person–culture match operates across multiple cultural layers (e.g., geographic, demographic, digital) and scales up to societal processes such as migration, personality development, cultural change, and social cohesion.

**What are the implications of the main findings?**

- Healthcare, public health, and policy interventions may be more effective when they consider sociocultural fit and perceived cultural alignment, rather than focusing solely on individual-level factors.
- Promoting sociocultural contexts that foster belonging, social recognition, and shared values can enhance well-being while also supporting social integration and societal resilience.

**Abstract:**

**Background**: Research on psychological well-being has traditionally focused on individual characteristics such as personality traits, values, and beliefs. However, comparatively less attention has been paid to the sociocultural contexts in which individuals are embedded and that influence how the individual characteristics are expressed, evaluated, and rewarded. One theoretical framework that captures this interaction is person–culture match (PCM), defined as the alignment between individual traits, values, or beliefs and those prevalent within the surrounding culture. **Objectives**: This narrative review synthesizes conceptual and empirical research on PCM and discusses its implications for psychological well-being and broader societal consequences. **Methods**: A narrative review of the literature was conducted to identify key theoretical contributions and empirical studies on PCM. The reviewed literature includes cross-cultural research examining the alignment between personal characteristics and corresponding cultural characteristics, as well as its implications for well-being and broader societal processes. **Results**: Across a wide range of studies, individuals tend to report higher well-being when their personal traits, values, or beliefs align with characteristics prevalent within their sociocultural context. This pattern has been documented across multiple characteristics, including personality traits, religiosity, political ideology, and personal values. Higher PCM has been associated with higher life satisfaction, greater positive affect, stronger self-esteem, and lower levels of stress and depressive symptoms. **Conclusions**: The literature suggests that well-being is shaped not only by individual characteristics but also by their alignment with one’s sociocultural contexts. Future research is needed to clarify the mechanisms underlying these effects and to explore the broader societal consequences of PCM. Considering cultural alignment may therefore be valuable for both advancing research and informing public health strategies and policy interventions aimed at enhancing well-being and social cohesion.

## 1. Introduction

Understanding what makes people happy is a central goal across disciplines, from psychology to sociology and public health. Research on well-being has traditionally emphasized individual characteristics such as personality traits, values, and beliefs as primary predictors of well-being [1,2]. However, individuals do not exist in isolation. Their traits and values are embedded in sociocultural contexts that shape how these characteristics are expressed, evaluated, and rewarded [3,4].

The concept of person–culture match (PCM) captures this interplay between individuals and their sociocultural context. Specifically, PCM refers to the alignment between a person’s traits, values, or beliefs and those prevalent in their sociocultural context [5,6]. Empirical evidence shows that individuals report higher well-being when their personal characteristics align with what is culturally common. This pattern has been documented across multiple match characteristics, well-being dimensions, and forms of culture. For example, individuals who prioritize security or civic virtue benefit more in countries where prosocial values are widespread [7,8], religious individuals report greater happiness and better health in religious states than in more secular ones [5,9], and individuals high in openness fare better in cities or neighborhoods that value openness on average as well [2,10]. Beyond its implications for individual well-being, research also suggests that the alignment between individuals and their sociocultural contexts may have broader societal consequences, for example, for processes of migration, cultural adaptation, and social integration.

### 1.1. Conceptual Foundations of Person–Culture Match

Person–culture match (PCM) refers to the extent to which an individual’s characteristics align with those that are common in their sociocultural context. PCM is characterized by three central features. First, PCM is trait-specific: alignment concerns the same characteristic at the individual and sociocultural level (e.g., personal religiosity and cultural religiosity [1,6]). Second, PCM depends on how widespread a trait, value, or belief is within a cultural context: Cultural characteristics refer to the aggregate level of a trait, value, or belief within a sociocultural context, indicating how widespread and socially normative it is. For example, religiosity is culturally prevalent when religious beliefs and practices are, on average, widely shared within a country or region [7,9]. Third, PCM reflects alignment rather than exact correspondence: A match does not require that an individual’s level of a specific characteristic exactly matches the cultural average; instead, individuals benefit from having traits, values, or beliefs that broadly align with what is culturally common. For example, a religious individual is considered to match their culture when people in their culture are, on average, religious, even if their personal religiosity does not exactly match the cultural average [1,5].

Several theoretical perspectives provide a foundation for why PCM matters for well-being. Belief congruence theory [11] and the similarity-attraction hypothesis [12] suggest that people experience greater satisfaction, interpersonal harmony, and social acceptance when their traits and values resemble those prevalent in their sociocultural context. Complementary perspectives, such as the self-enhancement theory [13] and terror management theory [14], emphasize that culturally supported characteristics help individuals maintain self-esteem and cope with existential concerns by affirming their value within a shared symbolic system.

Building on these theories, the well-being benefits of PCM can be understood as arising from basic psychological needs: self-validation and social belonging. When individuals share characteristics that are common in their culture, it signals that their self-concept is appropriate and provides a sense of psychological security [15,16,17]. Moreover, this alignment facilitates positive social interactions and fosters a sense of belonging within one’s sociocultural group, which supports emotional well-being and a stable sense of identity [18,19]. The reinforcement of these positive interactions and social rewards can further strengthen these effects, creating feedback loops that enhance the link between PCM and well-being. Although direct empirical tests of these processes are limited [1,20], this conceptual framework offers a plausible account of the positive association between PCM and well-being observed across studies.

### 1.2. Aim of the Review

The present article provides a narrative review of PCM research. The review aims to synthesize key conceptual perspectives and empirical findings, to highlight important boundary conditions, and to discuss broader societal and practical implications of PCM research. Rather than attempting an exhaustive or systematic review of all studies, it focuses on influential and frequently cited contributions that represent prevailing theoretical perspectives and empirical findings in the PCM literature. By integrating insights from multiple disciplines, this review underlines the relevance of PCM for both scientific research and practical efforts aimed at promoting well-being and improving societal outcomes, including migration, cultural adaptation, social cohesion, and public health.

The review is structured as follows. To start, the methodological approach of the narrative review is outlined, including the review design, literature search strategy, and study selection criteria. Section 3 then synthesizes empirical evidence on PCM, organized around key characteristics of PCM, its well-being consequences, conceptualizations of culture, and boundary conditions identified in prior research. Section 4 considers the broader societal consequences of PCM, including its role in migration patterns, personality development, cultural change, and social cohesion. Building on these insights, it further elaborates the practical implications of PCM for public health, policy, and applied settings. Finally, the review concludes with a summary of the main insights, the limitations of the present review, and directions for future research.

## 2. Methods

### 2.1. Review Design

The present article is a narrative (non-systematic) review, well suited for synthesizing research areas, integrating conceptual perspectives, and highlighting central empirical patterns. Rather than aiming for an exhaustive or fully systematic synthesis of the literature, this review provides a focused conceptual and empirical overview of PCM research and its links to well-being. As a narrative review, it does not follow a formal systematic review protocol. Instead, the review focuses on identifying central theoretical perspectives and representative empirical findings that have shaped the PCM literature, drawing on influential theoretical contributions and a diverse body of empirical research. In particular, the review examines how alignment between individual and sociocultural characteristics has been conceptualized, operationalized, and linked to well-being outcomes.

### 2.2. Search Strategy

Relevant literature was identified through structured searches in two major academic databases: PsycINFO (via EBSCO) and Web of Science. The search strategy combined terms related to person–culture match (e.g., “person–culture match,” “person–country match,” “person–region match,” “person–culture fit,” “person–country fit,” “person–region fit,” and “value similarity”) and terms referring to well-being outcomes (e.g., “well-being,” “mental health,” “life satisfaction,” “affect,” and “self-esteem”). These terms were selected to capture studies examining the alignment between individual and sociocultural characteristics and their association with well-being. The literature search primarily focused on publications from 2000 to 2025, reflecting the period in which PCM research has expanded substantially across psychology and related social sciences. As the PCM literature is still emerging, this review integrates established, widely cited research with more recent studies offering novel theoretical and empirical insights into the field.

### 2.3. Study Selection

To identify relevant studies, titles and abstracts were first screened for studies examining the alignment between individual-level characteristics and cultural-level characteristics (e.g., personality traits, values, beliefs) and their relationship with well-being. Full texts of potentially relevant studies were then reviewed in detail to confirm their suitability for inclusion.

Both theoretical and empirical peer-reviewed publications were considered. Studies were included when they (i) explicitly examined the alignment between individual-level characteristics and their cultural-level aggregates, (ii) focused on the match with sociocultural contexts, and (iii) addressed well-being or closely related psychological outcomes. Studies were excluded if they did not meet these criteria; no additional selection criteria were applied. This resulted in a final sample of 30 theoretical and empirical publications (for an overview, see Table 1). The selected studies were then examined and organized according to key conceptual and methodological features, including the type of match characteristics, the cultural level of analysis, and the well-being outcomes assessed. The study selection process involved database searches, title and abstract screening, full-text assessment, and final inclusion based on the predefined inclusion criteria.

## 3. Results: Empirical Evidence on Person–Culture Match and Well-Being

Across the reviewed literature, a consistent empirical pattern emerges: the relationship between personal characteristics and well-being is amplified when these characteristics are prevalent within the surrounding sociocultural context. Rather than exerting uniform main effects, empirical findings consistently indicate that the strength of the relationship between personal characteristics and well-being depends on how widely the characteristics are shared within a culture. Initial evidence for this interactional pattern stems from cross-cultural research by Fulmer and colleagues [2]. Analyzing more than 7000 respondents across 28 countries, the authors demonstrated that the associations between personality traits (e.g., extraversion, promotion focus) and well-being were stronger in cultures where these traits were culturally prevalent. Individuals thus appear to benefit psychologically when their personal characteristics align with those commonly found in their culture. Building on this seminal work, subsequent studies have replicated and extended these findings across a broad range of match characteristics, well-being dimensions, and cultural levels (see Table 1).

### 3.1. Match Characteristics

Accumulating evidence indicates that PCM effects on well-being generalize across a wide range of characteristics, establishing PCM as a robust empirical phenomenon rather than a domain-specific exception. For clarity, the evidence can be grouped into personality traits, values, religiosity, political ideology, and material or lifestyle-related characteristics (see Table 1).

In the realm of personality, large-scale cross-cultural research demonstrates that traits such as openness and conscientiousness are associated with higher well-being in cultural contexts where these traits are common [10,21]. Importantly, however, these effects are trait-specific rather than universal across the Big Five, as only some traits exhibit consistent PCM effects while others do not [10,21,22]. Recent cross-cultural evidence further indicates that personality-based PCM effects extend beyond the Big Five, with agentic individuals reporting higher life satisfaction in cultures characterized by higher aggregate agency and greater cultural homogeneity [41].

Research on basic human values reveals similar PCM patterns. Individuals who prioritize conformity and security report better well-being when living in cultures that endorse these values [8,26]. Importantly, value match effects are not uniform but depend on the content of the values themselves. For example, alignment in some values tends to enhance well-being, whereas alignment in other values may reduce or even decrease well-being in certain cultural contexts [8,24,27]. Beyond these basic values, PCM effects have also been observed for civic and prosocial values, with helping behavior and civic engagement promoting higher well-being primarily in cultures where such behaviors are socially normative [7,23]. Beyond cultural alignment and for a broader discussion, research on value congruence has a long tradition beyond cultural alignment, including person-group fit in universities [42,43], classrooms [44,45], peer groups [46], and close social networks [47,48]. Moreover, value congruence promotes well-being also within families and intimate partnerships, highlighting multi-layered fit processes. These findings, however, exceed cultural alignment and differ in how value similarity is conceptualized, measured, and operationalized.

Religiosity represents the most extensively documented and most robust match characteristic. Across numerous studies, religious individuals consistently report higher well-being in cultures where religiosity is widespread than in more secular cultures [1,5,6,9,20,29,30,32,34]. This pattern extends beyond psychological and social well-being to indicators of physical health and greater longevity [9,30]—highlighting the breadth of the PCM effects on well-being.

Beyond religiosity, PCM effects are also observed for political and ideological beliefs. For example, conservatives report higher happiness and life satisfaction when living in culturally conservative sociocultural contexts [35]. Again, these ideological match effects can even extend to physical health outcomes: partisans tend to live longer when residing among like-minded people [36]. Similar patterns emerge for broader worldviews. For instance, the well-being benefits of believing in scientific and technological progress are amplified when such beliefs are widely shared within a culture [37].

Moreover, PCM effects on well-being are not confined to psychological traits, values, or beliefs. They also extend to material and lifestyle-related characteristics. For example, the relationship between income and subjective well-being is stronger in wealthier cultures than in poorer ones, suggesting that economic resources confer greater social and psychological benefits when they align with societal standards of living [38]. Similarly, the impact of marital status on life satisfaction depends on cultural context: the more common marriage is within a culture, the stronger its positive association with individual well-being [40]. This pattern also applies to physical characteristics: obese individuals reported higher life satisfaction in sociocultural contexts where obesity was more prevalent, potentially highlighting the role of normative comparison processes in physical self-evaluation [39].

### 3.2. Well-Being Consequences of PCM

Beyond the question of which personal characteristics matter, an equally important question concerns which well-being dimensions are associated with PCM. Although the strength of PCM effects may vary across specific well-being dimensions [6,8,49], PCM-related benefits span emotional, cognitive, social, and physical well-being. Individuals with greater cultural alignment report more positive and fewer negative emotions [1,8,22,28,38], greater life satisfaction [7,8,10,20,21,32], and higher self-esteem [2,5,8,10,26,33], as well as stronger social well-being, including supportive relationships, feelings of respect, and a sense of belonging [1,6,8,20,22]. Notably, physical health is also improved, with links to reduced depressive symptoms [26,50], better subjective health [6,8,31,39], and even increased longevity [10,30,36]. Collectively, these findings indicate that PCM contributes to multiple dimensions of well-being, supporting psychological, social, and physical health in ways that may accumulate over time.

### 3.3. Conceptualization of Culture

Across this large body of research, most PCM studies have traditionally equated culture with geographic units such as countries, regions, or federal states. More recent research, however, challenges this narrow conceptualization and conceptualizes culture as a system of shared values, traits, and behaviors that are transmitted across time and generations, distinguishing one cultural group from another [51,52].

Indeed, PCM operates not only at national and regional levels but also within demographic cultural units. Vogel et al. (2026) demonstrated that PCM also operates within geo-demographic cultures [50]. Using data from more than 850,000 individuals in the United States, the authors showed that match effects for religiosity and political orientation were not limited to federal states but extended to demographic groups—particularly ethnicity and gender. For example, individuals reported higher well-being when their ideological orientations aligned with those prevalent both in their federal state and within their ethnic group. These findings suggest that culture is most influential when it captures both where people live and who they are.

Consistent with this geo-demographic perspective of culture, subsequent research has identified religion as another powerful cultural context for PCM [28]. In particular, the match to one’s own fine-grained religious group within one’s country appears to drive the well-being benefits of a match. Together, these studies broaden the conceptual scope of PCM, demonstrating that culture is a multilayered system of shared meanings, norms, and identities that extends beyond geographic boundaries.

### 3.4. Boundary Conditions and Limits of PCM

Although the empirical literature provides robust evidence that PCM is associated with well-being across multiple match characteristics, well-being dimensions, and forms of culture, these effects are not universal. In the domains of personality [10,21,22] and values [8,24,27], match effects have been observed for some characteristics but not others. These findings suggest that cultural alignment is consequential in many—but not all—domains, and that the conditions under which match matters remain not fully understood. In addition to these conceptual boundary conditions, the PCM literature is also unevenly distributed across global regions. Much of the existing research relies on samples from Western and industrialized contexts, whereas regions such as Africa, South Asia, and Oceania remain underrepresented. This may limit the generalizability of the current evidence base, and future research should extend PCM investigations to more diverse cultural contexts. Taken together, these considerations highlight several important boundary conditions of PCM effects.

First, PCM effects appear asymmetrical and dependent on the match characteristic. For instance, while religious individuals benefit psychologically in highly religious contexts, non-religious individuals in secular cultures often show little to no PCM advantage. Similarly, political match effects are more pronounced for conservatives in culturally conservative regions than for liberals in liberal regions, possibly reflecting differences in visibility and social reinforcement of these traits [35,36].

Second, PCM effects do not appear to be equally relevant for all individuals. PCM tends to be particularly relevant for individuals who are socially oriented and for whom relationships, group belonging, and social approval are central concerns. For these individuals, PCM tends to be associated with higher well-being. In contrast, for more individualistic or autonomy-focused individuals, cultural alignment may be less beneficial or even detrimental [33]. Moreover, PCM promotes well-being primarily when individuals perceive their culture as central to their self-concept and identify strongly with it. Individuals for whom cultural group membership is peripheral gain little or no benefit from cultural alignment [28].

Third, not all sociocultural contexts are equally relevant for PCM. Match effects appear strongest when the cultural context is clearly defined, socially salient, and personally meaningful. For example, PCM with demographic groups such as religious communities, ethnic groups, or gender-based cultures tends to be more consequential than alignment with broader sociocultural groups [28,50]. These differences highlight that PCM may depend on how strongly individuals identify with a given sociocultural group and how important that group is for their sense of self.

Fourth, PCM must be psychologically perceived to translate into well-being benefits. Recent evidence suggests that objectively matching to the prevalent characteristics within one’s culture is unlikely to translate into well-being benefits if individuals are unaware of this match [6]. Instead, subjective PCM—matching to the perceived characteristics of one’s culture—seems to be a critical pathway through which PCM affects well-being. Accordingly, interventions focusing solely on structural or demographic alignment may be insufficient unless they also foster individuals’ awareness of belonging, acceptance, and perception of match. Together, these boundary conditions underscore that PCM is not a one-size-fits-all solution. Its relevance depends on who the individual is, which cultural groups are involved, and whether cultural alignment is experienced and socially recognized in everyday life. Recognizing these limits is essential for developing effective public health strategies and policy interventions that seek to leverage cultural alignment to improve well-being. Importantly, beyond individual well-being, emerging research suggests that PCM may also shape broader societal processes, such as migration patterns or social cohesion.

## 4. Discussion

### 4.1. Summary of Well-Being Findings

Across diverse domains and methodological approaches, the reviewed literature reveals a consistent interactional pattern: the relationship between individual characteristics and well-being depends systematically on their prevalence in the surrounding sociocultural context. Rather than exerting uniform main effects, personal traits, values, and beliefs appear to be psychologically beneficial, particularly when they align with culturally shared norms and characteristics.

This pattern has been documented across a wide range of match characteristics, including personality traits, personal values, religiosity, political ideology, and even lifestyle-related factors such as income, marital status, and physical characteristics. Moreover, the consequences of PCM extend across multiple dimensions of well-being, encompassing emotional experiences, cognitive evaluations of life satisfaction, self-related outcomes such as self-esteem, vulnerability to stress and depression, and even physical health and longevity.

At the same time, the literature highlights important boundary conditions. PCM appears to be most consequential for individuals who strongly identify with their sociocultural context and for cultural groups that are socially salient and personally meaningful. Furthermore, subjective perceptions of cultural alignment often matter more than objective cultural alignment, underscoring the psychological nature of PCM processes.

Taken together, these findings suggest that well-being is not solely a product of individual characteristics but emerges from the dynamic interplay between persons and their sociocultural environments. While the primary literature search focused on empirical studies linking PCM to well-being outcomes, additional theoretical and empirical contributions were identified through citation tracking and conceptual integration of adjacent research areas. These studies extend the implications of PCM beyond individual well-being and illustrate how alignment processes scale to broader societal dynamics.

### 4.2. Societal Consequences of Person–Culture Match

By shaping where people choose to live, how they adapt to their environments, and how cultures evolve over time, alignment between individual characteristics and sociocultural contexts plays a central role in broader societal processes. In this section, we examine these societal consequences of PCM, focusing on migration patterns, personality development, intergroup relations, and cultural change.

#### 4.2.1. Migration Patterns

One of the most visible consequences of PCM is its impact on migration patterns. Individuals do not relocate randomly; rather, they tend to move toward environments that align more closely with their traits, values, and beliefs. This phenomenon occurs not only at the level of international migration but also locally, as people sort into neighborhoods or communities that reflect their political, religious, or moral orientations. For example, individuals high in sociability are more likely to move to regions characterized by frequent and diverse social interactions, whereas those high in emotionality may prefer smaller, more familiar regions that better align with their emotional needs [53]. Similarly, Motyl et al. (2014) found that individuals are more likely to relocate to communities where their political or moral beliefs are widely shared and report a stronger sense of belonging in such contexts [54]. This stronger sense of belonging from PCM extends to immigrants, who are more likely to identify with their host society and experience enhanced well-being when their personal characteristics align with the cultural context [55]. Such alignment facilitates smoother integration into new communities.

Although these self-selection processes can enhance individual well-being by increasing PCM, they may simultaneously contribute to ideological clustering and polarization. From a societal perspective, PCM may thus help explain both adaptive migration patterns and the emergence of ideological segregation, potentially deepening societal divides.

#### 4.2.2. Personality Development and Cultural Adaptation

PCM also has profound implications for personality development and the ways in which individuals adapt to their sociocultural contexts over time. Longitudinal research suggests that individuals whose personality traits align well with their sociocultural context exhibit greater personality stability over time, whereas those experiencing misalignment are more likely to undergo adaptive personality change [56]. More recent work further highlights the bidirectional nature of these processes. Entringer et al. (2023) showed that personality traits and religious cultures mutually influence each other over time [57]. Individuals may gradually adjust traits such as agreeableness or openness as they adapt to prevailing sociocultural norms, while aggregated individual changes can, in turn, reshape cultural profiles. These findings underscore that PCM is not static but embedded in dynamic processes of mutual adjustment between individuals and their sociocultural contexts. Extending these findings to migration contexts, recent work shows that alignment between personal characteristics and host cultural norms is associated with better psychological well-being among immigrants, highlighting the role of PCM in supporting adaptation to new sociocultural environments [58].

The influence of PCM on personality development can also be understood through the lens of cultural evolution. As individuals are embedded in—and interact with—different sociocultural contexts, they may undergo personality changes that enhance their ability to adapt and function effectively within these sociocultural contexts. This perspective is consistent with the concept of balancing selection in evolutionary psychology, which suggests that individuals whose traits fit well with their cultural environments may experience adaptive advantages [59].

#### 4.2.3. Cultural Change

At a macro level, PCM contributes to both cultural continuity and cultural change. Cultures are not static but evolve in response to ecological, economic, and technological shifts [60]. The concept of PCM helps explain how such cultural evolution unfolds over time. Individuals whose characteristics align with emerging cultural norms are more likely to thrive, whereas those experiencing sustained mismatch may disengage, relocate, or advocate for change. Over time, these alignment processes—arising from the interplay between personal traits and sociocultural contexts—can reinforce existing cultural trends or accelerate cultural transformation, as individual preferences and behaviors accumulate and spread throughout society. Accordingly, the process of cultural evolution can be understood as a dynamic process of adaptation in which PCM plays a central role.

#### 4.2.4. Social Cohesion and Intergroup Attitudes

Another critical societal implication of PCM concerns social cohesion and intergroup attitudes. When individuals perceive their values and behaviors as congruent with those around them, they are more likely to develop positive attitudes toward outgroups, thereby reducing prejudice, fostering social integration, and contributing to a cohesive and resilient society [25]. Furthermore, value congruence has been shown to strengthen national pride, suggesting that alignment between personal and collective values not only enhances individual well-being but also reinforces identification with larger sociocultural groups [61]. This dynamic is particularly important in multicultural contexts, where PCM can help bridge divides and promote inclusion by facilitating positive intercultural interactions and reducing tensions across different sociocultural groups. Conversely, misalignment can disrupt social cohesion. For instance, individuals are more likely to reduce social ties and unfollow others on social media when perceived values differ [62].

Taken together, the influence of PCM is not confined to the personal sphere but extends throughout society, shaping migration patterns, personality development, cultural change, and social cohesion. By linking individual-level alignment processes to broader societal dynamics, this perspective highlights how PCM scales up to influence collective outcomes that are directly relevant for public health, social integration, and societal stability.

### 4.3. Practical Implications of Person–Culture Match Research

Building on these individual- and societal-level dynamics, PCM research offers valuable insights for healthcare, public policy, education, and organizational practice. In an increasingly interconnected and rapidly changing world—characterized by globalization, digitalization, and high mobility—understanding PCM can inform practical interventions aimed at promoting well-being, social integration, and societal resilience across diverse sociocultural contexts.

#### 4.3.1. Well-Being and Public Health Interventions

From a clinical perspective, PCM can be understood as an important factor contributing to well-being. Individuals whose personal characteristics align with the prevalent traits, values, or beliefs in their sociocultural context are better positioned to thrive. Conversely, cultural mismatch can represent an underrecognized source of psychological distress. Individuals whose traits, values, or beliefs are not widely represented in their sociocultural context may experience chronic stress, alienation, or reduced self-worth. Mental health professionals can integrate PCM-informed interventions to strengthen well-being. This includes supporting identity coherence, addressing perceptions of cultural alignment, and facilitating connections with value-congruent communities—both in person and through online networks. For example, therapists may explicitly explore whether clients experience a mismatch between their personal values (e.g., autonomy, religiosity, or lifestyle preferences) and those dominant in their sociocultural environment. They may then work to strengthen coping strategies or help clients connect with more congruent sociocultural contexts. Such interventions may enhance feelings of belonging and psychological security, complementing established therapeutic strategies.Beyond individual therapeutic approaches, PCM also offers valuable insights for public health initiatives and health policy. Health campaigns, such as those promoting exercise or nutrition, may be more effective when framed to align with culturally prevalent values, increasing engagement and adherence. For instance, public health campaigns promoting physical activity may emphasize individual achievement and self-optimization in more individualistic contexts, whereas the same campaigns may highlight social responsibility and family well-being in more collectivistic cultural environments. Similarly, policies aimed at improving mental health services can be tailored to the cultural profiles of diverse populations, fostering inclusivity and addressing group-specific needs. For example, religious individuals living in increasingly secular societies may benefit from programs that connect them with like-minded sociocultural contexts. These culturally sensitive interventions can help mitigate feelings of disconnection, promote a sense of belonging, and improve both engagement and treatment outcomes.

#### 4.3.2. Intergroup Relations and Social Cohesion

Especially in today’s polarized and fragmented societies, PCM-informed strategies can guide the design of integration and social cohesion initiatives. PCM is facilitated not only through interpersonal contact but also through a perceived alignment between individuals’ values and the broader sociocultural environment. A sense of cultural match that extends beyond immediate sociocultural in-groups to shared civic values can help bridge social divides and mitigate ideological polarization. Recognizing a shared framework of traits, values, and beliefs has the potential to improve intergroup relations, reduce prejudice, and foster inclusive environments in which individuals feel that their values are acknowledged and respected.

In practice, this may involve designing integration programs that emphasize shared civic principles (e.g., mutual respect, democratic values) while allowing for cultural diversity in their expression. Community-based dialogue formats or structured intergroup contact interventions, for example, can highlight both commonalities and differences in a constructive manner. Such approaches may be especially beneficial for marginalized groups, including individuals from ethnic, religious, or socioeconomically disadvantaged backgrounds, who often feel disconnected from dominant societal narratives. In addition, when individuals perceive that their core values and traits are represented in collective narratives, they are more likely to develop trust in social institutions and to engage cooperatively with others. Facilitating cultural match at this broader societal level can therefore foster positive intergroup attitudes and social cohesion.

#### 4.3.3. Digital Communities and Online Contexts of PCM

Digitalization and social media introduce new contexts for PCM by enabling individuals to self-select into online communities based on shared values, beliefs, or identities. For individuals whose personal characteristics are not widely represented in their immediate geographic environment, digital spaces can provide important sources of cultural alignment, social support, and well-being. From a public health and societal perspective, digitally facilitated PCM may thus enhance individual well-being through an increased sense of belonging.

At the same time, cultural alignment may undermine social cohesion when it occurs primarily within ideologically homogeneous online spaces. When alignment is achieved exclusively within such homogeneous environments, opportunities for constructive cross-group interaction may diminish, potentially reducing the broader positive outcomes of PCM and reinforcing ideological echo chambers, polarization, and social fragmentation. From a practical perspective, platform designers and policymakers can mitigate these risks by promoting algorithmic diversity (e.g., exposing users to a broader range of perspectives) and by supporting digital interventions that encourage constructive cross-group dialogue rather than purely similarity-based clustering. Promoting online environments that support well-being while encouraging exposure to diverse perspectives may help realize the benefits of digital cultural alignment without exacerbating societal divides.

#### 4.3.4. Educational and Organizational Settings of PCM

Finally, PCM principles are equally applicable in educational and organizational settings. Learning environments that reflect students’ values and personal characteristics can foster engagement, academic success, and socioemotional development—factors closely linked to long-term well-being and mental health. For example, educators may implement flexible curricula that allow students to connect learning content to their own cultural backgrounds or personal values, or create classroom environments that explicitly recognize diverse perspectives.

Similarly, workplaces that align organizational culture with employees’ values and personal characteristics may enhance job satisfaction, engagement, and psychological well-being. In practice, this may include value-based recruitment processes, organizational culture assessments, or the creation of employee resource groups that support alignment between individual and organizational values. By acknowledging cultural diversity and supporting cultural alignment, educational and organizational institutions can create environments that promote both individual well-being and collective effectiveness.

#### 4.3.5. Practical Principles Across Domains

Across all settings discussed above, several general principles emerge for designing PCM-informed interventions and policies. First, interventions should consider both the individual and the cultural environment, recognizing that well-being emerges from their interplay. Second, perceived cultural alignment often matters more than objective cultural alignment, highlighting the importance of awareness, communication, and subjective experience. Third, the relevance and salience of the cultural group must be considered, as alignment is most beneficial when the group is meaningful to the individual. Finally, PCM-informed strategies should remain adaptive, acknowledging that both cultural contexts and personal traits are dynamic and mutually influencing over time. Together, these principles provide a practical framework for applying PCM research in healthcare, public policy, digital environments, as well as educational and organizational practice, ultimately fostering well-being, social cohesion, and societal resilience, while maintaining sensitivity to cultural diversity and change.

## 5. Conclusions

Person–culture match provides a useful but still developing conceptual framework for understanding how individual well-being is embedded within sociocultural contexts. The empirical evidence reviewed here suggests that well-being is shaped not only by personal characteristics but also by the extent to which these characteristics align with the surrounding sociocultural context. At the same time, the strength and consistency of these associations vary across match characteristics, well-being components, and cultural contexts, highlighting important methodological and contextual differences within the literature. By shaping migration decisions, personality development, cultural change, and social cohesion, PCM links psychological processes to broader societal outcomes. Integrating PCM insights into healthcare, policy, education, and organizational practice may thus offer valuable perspectives for addressing key societal challenges and promoting more inclusive and healthy societies. However, further research is needed to clarify the conditions under which such applications are most effective.

## Figures and Tables

**Table 1:** Illustrative overview of empirical studies examining person–culture match across different cultural contexts, match characteristics, and well-being outcomes.

Study	Match Characteristic	Well-being Outcome	Sociocultural Context
Personality			
Fulmer et al. (2010) [2]	Extraversion, Promotion focus, Locomotion	Life satisfaction, Positive emotions, Life happiness, Self-esteem	Countries
Jokela et al. (2015) [21]	Big Five traits	Life satisfaction	Postal districts of London metropolitan area
Bleidorn et al. (2016) [10]	Big Five traits; Religiosity	Self-esteem	Cities across the 50 U.S. states
Götz et al. (2018) [22]	Big Five traits	Life satisfaction, Satisfaction with personal relationships, Positive affect, Negative affect	Swiss cantons
**Values**			
Stavrova, Schlösser, Fetchenhauer (2013) [7]	Civic virtue	Happiness, life satisfaction	Countries
Oarga et al. (2015) [23]	Civic engagement values	Life satisfaction, Positive affect	Countries
Van Den Broeck et al. (2019) [24]	Values	Happiness, Life satisfaction, Perceived health	Countries
Hanel et al. (2020) [8]	Values	Life satisfaction/happiness, Affect, Self-esteem, Physical well-being, Social well-being, Supportive Relationship	Countries; regions
Wolf et al. (2021) [25]	Values	Life satisfaction	Fellow citizens
Du et al. (2023) [26]	Values	Self-esteem	U.S. federal states; U.S. cities
Kirchner-Häusler et al. (2024) [27]	Honor values	Evaluation of nine life domains; Life satisfaction	Countries
Vogel et al. (2026) [28]	Values	Affect, Life satisfaction, Self-esteem	Religious cultures in Germany
**Religiosity**			
Diener et al. (2011) [1]	Religiosity	Subjective well-being	Countries
Eichhorn (2012) [29]	Religiosity	Life satisfaction	Countries
Stavrova, Fetchenhauer & Schlösser (2013) [20]	Religiosity	Happiness, Life satisfaction	Countries
Stavrova (2015) [30]	Religiosity	Physical health	Countries (Study 1); U.S. regions (Study 2)
Gebauer et al. (2017) [5]	Religiosity	Self-esteem	Countries
Stroope & Baker (2018) [31]	Religiosity	(Self-rated) Health	U.S. counties
Pöhls et al. (2020) [32]	Religiosity	Life satisfaction	Countries
Gebauer et al. (2020) [33]	Religiosity; Political orientation (Supplement)	Self-esteem; Depression (Supplement)	Countries; U.S. federal states (Supplement)
Ebert et al. (2020) [9]	Religiosity	Longevity	U.S. counties
Ugur & Aydin (2023) [34]	Religiosity	Happiness	Provinces in Turkey
Vogel et al. (2025) [6]	Religiosity	Global well-being; Psychological well-being (life satisfaction, self-esteem, affect), Physical well-being, Social well-being	Countries
**Political and Ideological Beliefs**			
Stavrova & Luhmann (2016) [35]	Political orientation	Happiness, Life satisfaction	Specific time periods in the U.S. (Study 1); Countries (Study 2)
Ebert et al. (2023) [36]	Political ideology	Longevity	U.S. counties
Vogel et al. (2026) [28]	Religiosity, Political orientation	Well-being	U.S. federal states and demographic groups
Stavrova et al. (2016) [37]	Belief in scientific–technological progress	Life satisfaction	Countries
**Material or Lifestyle Characteristics**			
Tay et al. (2014) [38]	Income	Life evaluation, Positive affect, Negative affect	Countries
Wadsworth & Pendergast (2014) [39]	Body weight/obesity prevalence	Life satisfaction	U.S. counties
Wadsworth (2016) [40]	Marriage status	Life satisfaction	Demographic characteristics

## Data Availability

This article is a review of existing literature, and no new datasets were generated or analyzed during the study. For access to the datasets used in the studies referenced throughout the manuscript, please refer to the original publications.

## Abbreviations

The following abbreviations are used in this manuscript:

PCM	Person–culture match
